# Diversity, classification, and evolution of myxobacterial PilY1 proteins

**DOI:** 10.3389/fmicb.2026.1826482

**Published:** 2026-05-07

**Authors:** Utkarsha Mahanta, Roman Waßmuth, Sherin Brighty, Anke Treuner-Lange, Gaurav Sharma

**Affiliations:** 1 Department of Biotechnology, Indian Institute of Technology Hyderabad, Sangareddy, Telangana, India; 2 Max Planck Institute for Terrestrial Microbiology, Marburg, Germany

**Keywords:** adhesion, minor pilin/PilY1 complexes, PilY1 proteins, surface motility, type IVa pili

## Abstract

Type IVa pili (T4aP) mediate one of the most widespread forms of bacterial surface motility through coordinated cycles of extension, attachment, and retraction that generate pulling forces to propel cells forward. This process is well characterized in diverse Gram-negative bacteria such as *Pseudomonas*, *Myxococcus*, and *Neisseria*, where T4aP filaments are composed of thousands of major pilin subunits and a tip complex formed by minor pilins and the PilY1 adhesin proteins. PilY1 is a multifunctional protein localized at the T4aP machine and pilus tip, playing critical roles in pilus priming, surface adhesion, motility, and virulence. *Myxococcus xanthus* possesses three distinct PilY1 adhesins with conserved C-terminal but different N-terminal, where each is encoded within separate minor pilin/*pilY1* gene clusters, suggesting functional specialization. This study investigates the extent of PilY1 diversity and domain architecture conservation across the phylum Myxococcota using genomic, phylogenetic, and structural approaches, suggesting a remarkable evolutionary strategy for tailoring T4aP tip complexes to diverse environmental and physiological demands. Our analysis of sixty-seven representative genomes reveals that PilY1 proteins are widely distributed and typically occur in multiple copies, with an average of two homologs per genome. Phylogenetic reconstruction identifies several well-supported clades supported by myxobacterial taxonomy, domain architecture, protein length, and cysteine content. Notably, *M. xanthus* paralogs PilY1.1 and PilY1.2 form a conserved lineage characterized by a DUF4114 domain and appear to have evolved primarily through vertical inheritance, whereas PilY1.3 clusters with homologs from diverse bacterial phyla, suggesting acquisition via horizontal gene transfer. We reconfirmed that *pilY1* genes frequently occur in conserved operons with minor pilins (*pilX*, *pilW*, *pilV*, and *fimU*), supporting their role in forming priming complexes initiating pilus assembly. Structural modeling predicts conserved interaction patterns within minor pilins and PilY1 via β-strand complementation between PilX and PilY1, highlighting a potentially conserved structural feature of T4aP tip complexes. Together, our findings reveal extensive diversification of PilY1 proteins within Myxococcota and suggest that variation in their N-terminal domains contributes to functional specialization of T4aP systems. Future experimental studies will be essential to determine how this diversity shapes mechanosensing, adhesion, and environmental adaptation in myxobacteria and other bacteria.

## 1 Introduction

Myxobacteria are Gram-negative soil bacteria renowned for their complex social behaviors, cooperative predation, and multicellular developmental cycles. Efficient surface motility is central to these collective behaviors, which is well-known in model organism *Myxococcus xanthus* (Kroos et al., 2025). Their social motility is largely mediated by type IVa pili (T4aP), which are dynamic filamentous appendages, made up of major pilins, minor pilins and a PilY1 adhesin, that extend, attach to substrates, and retract to generate pulling forces that propel cells across surfaces (Sharma et al., 2018a,Treuner-Lange et al., 2020, Kroos et al., 2025). This well-conserved machinery enables coordinated swarm movement and interactions with extracellular polysaccharides referred to as EPS, facilitating predation and multicellular development (Perez-Burgos and Søgaard-Andersen, 2020, Kroos et al., 2025). A key component of the T4aP tip complex is the adhesin PilY1, which is thought to mediate surface attachment, mechanosensing, and regulation of pilus activity (Treuner-Lange et al., 2020, Herfurth et al., 2022, Xue et al., 2022, Kroos et al., 2025).

The discovery of PilY1, known as PilC in Neisseria, dates back to 1991, when Jonsson, Nyberg, and Normark identified a novel outer membrane protein, PilC, that co-purified with the major pilin PilE in highly purified T4aP preparations from *N. gonorrhoeae* strain MS11 (Jonsson et al., 1991). Loss of PilC expression abolished pilus assembly, establishing its essential role in T4aP biogenesis. This strain encodes two non-identical PilC paralogs (PilC1 and PilC2), whose expression is regulated by translational phase variation via slipped-strand mispairing in poly-G tracts within their signal peptide-encoding regions, a mechanism enabling immune evasion (Jonsson et al., 1991). Functional studies confirmed that PilC proteins are critical for adhesion to human epithelial cells (Rudel et al., 1992, Nassif et al., 1994). Overexpression of PilC2-His_6_ from *N. gonorrhoeae* not only bound to human cells but also blocked adherence of piliated wild-type bacteria, suggesting a competitive inhibition mechanism (Rudel et al., 1995). Immunogold labeling localized PilC to the tip of the pilus, identifying it as a T4aP tip-located adhesin, a pivotal finding that redefined the functional role of pilus tips (Rudel et al., 1995). Interestingly, sequence differences in the N-terminal domains of PilC1 and PilC2 from *N. meningitidis* underlie their functional divergence: only PilC1, but not PilC2, contributes to modulation of bacterial adhesiveness (Morand et al., 2001). The *P. aeruginosa* homolog, PilY1 was identified as a C-terminal homolog of Neisseria PilC encoded within a gene cluster containing six minor pilins (Alm et al., 1996). Like its Neisseria counterpart, PilY1 was detected in both the outer membrane and pilus fractions (Alm et al., 1996). A major milestone was the structural and functional analysis of the C-terminal domain of *P. aeruginosa* PilY1 (residues 591–1161), crystallized at 2.1 Å resolution which revealed a seven-bladed β-propeller fold and a distinct EF-hand-like calcium-binding site (Orans et al., 2010), therefore, defining the PFAM domain PF05567 (PilY1 beta-propeller domain). Functional studies showed that a D859A mutation (disrupting calcium binding) abolished T4aP formation, while a D859K mutation (mimicking calcium-bound state) produced non-functional pili, phenocopying a non-retractile pilT deletion (Orans et al., 2010). These findings led to the model that PilY1 acts as a calcium-regulated switch, controlling the balance between pilus extension and retraction. The calcium-binding motif of *P. aeruginosa* PilY1 [motif 1, Dx(D/N)xDGxxD] was also identified in PilC1 and PilC2 of *N. gonorrhoeae* and *N. meningitidis* (Orans et al., 2010). Functional validation in *N. gonorrhoeae* showed that a calcium-binding-deficient PilC1 failed to support adhesion, while a truncated C-terminal PilC1 did not block adherence, indicating that the C-terminal domain is not involved in adhesion (Cheng et al., 2013). PilY1 was also shown to bind integrins in a calcium-dependent manner with an identified RGD motif (aa 619–621) critical for this interaction (Johnson et al., 2011). This motif lies near a second calcium-binding site (DxDxNxxxD, aa 600–608), where a D_608_A mutation abolished integrin binding, while D_608_K preserved it, highlighting the dual role of calcium in ligand recognition (Johnson et al., 2011). In *Kingella kingae*, two PilC-like proteins (PilC1 and PilC2) regulate T4aP-dependent motility and adherence. While T4aP formation requires either PilC1 or PilC2, their roles diverge; while PilC1 contains a canonical calcium-binding motif and is essential for motility and adherence, PilC2 has a 12-aa motif homologous to calmodulin, suggesting a distinct calcium-sensing mechanism (Porsch et al., 2013). Calcium binding was not required for T4aP formation but was essential for PilC1-mediated motility and adherence, and partially required for PilC2-mediated motility, underscoring functional specialization (Porsch et al., 2013). The plant-pathogenic bacterium *Xylella fastidiosa*, whose T4aP-dependent motility is enhanced by elevated calcium concentrations, encodes three distinct PilY1 proteins, with only PilY1.1 (PD1611) containing a canonical calcium-binding motif 1 (Cruz et al., 2014). Although PilY1.1 is not essential for T4aP formation, the calcium-stimulated enhancement of motility is lost in its absence (Cruz et al., 2014). A genome-wide survey of PilY1 homologs in plant-pathogenic bacteria revealed that strains harbor one to three PilY1 proteins, each varying in calcium-binding motif content: some lack motifs entirely, others contain one, or combinations of motifs 1 and 2, or 1 and 3 (motif 3, DxD/NxDxxxxxxD/E) (Cruz et al., 2014).

The next milestone was the discovery that in *P. aeruginosa* minor pilins not only prime T4aP assembly but are also required for cell surface localization of the pilus-associated PilY1 protein, strongly indicating complex formation between minor pilins and PilY1 (Nguyen et al., 2015c). Minor pilins, low-abundance proteins structurally resembling major pilins, are known from T4aP systems and homologous Type 2 secretion systems (T2SS) and are differentiated into core and non-core types (Giltner et al., 2012). In *P. aeruginosa* four core minor pilins (FimU, PilV, PilW and PilX) and one non-core minor pilin (PilE) are known, while in *Neisseria*, core minor pilins are called PilH, PilI, PilJ, PilK and non-core minor pilins are called PilX/L, PilV and ComP (Giltner et al., 2012). Functional analyses in *P. aeruginosa* indicate distinct roles and dependencies; while PilV, PilW, and PilX and PilY1 depend on each other for pilus-association, PilE requires PilVWXY1 for its pilus-incorporation and FimU is incorporated independently (Nguyen et al., 2015c). Interactions between core and non-core minor pilins further support complex formation (Nguyen et al., 2015b,Nguyen et al., 2015c).

The first N-terminal domain of PilY1 proteins was described in 2017 by Hoppe et al. (Hoppe et al., 2017). Deletion of the single PilY1 protein from *Legionella pneumophila* caused defects in T4aP-dependent motility and host cell adherence (Hoppe et al., 2017). Interestingly, these authors identified an N-terminal domain with homology to the von Willebrand factor A (vWFA; also known as VWA) domain, and found similar homologous domains in other PilY1 proteins, suggesting a role in mechanosensing (Hoppe et al., 2017). In *P. aeruginosa*, deletion of the vWFA domain reduced T4aP formation and motility, and a single cysteine residue within it was critical for surface signaling suggesting disulfide bridge formation may be involved in force sensing (Webster et al., 2022). Notably, this vWFA domain also contains a conserved metal-ion-dependent adhesion site (MIDAS, DxSxS.T…D) suggested to be involved in adherence (Webster et al., 2022).

The predatory soil bacterium *M. xanthus* encodes three distinct PilY1 proteins (PilY1.1, PilY1.2, PilY1.3), each located within separate minor pilin/*pilY1* gene clusters (cluster 1–3) (Treuner-Lange et al., 2020). While all share the conserved C-terminal β-propeller domain, their N-terminal domains vary significantly. PilY1.3 contains a vWFA domain while PilY1.1 and PilY1.2 contain a DUF4114 domain (Treuner-Lange et al., 2020, Herfurth et al., 2022, Xue et al., 2022). Functional studies revealed that in *M. xanthus* either PilY1.1 alone or PilY1.3 alone (cluster 1 or 3 respectively) is sufficient for T4aP formation and motility under vegetative growth and that a deletion of the vWFA domain in PilY1.3 impaired T4aP formation and motility in the absence of PilY1.1 and PilY1.2 (Treuner-Lange et al., 2020). Cryo-electron tomography (Cryo-ET) revealed that the minor pilins and PilY1 form a priming complex extending from the inner membrane up to the periplasmic cavity of the secretin pore (Treuner-Lange et al., 2020). Also, minor pilins and PilY1 proteins from *M. xanthus* were found in pure T4aP pilus preparations (Treuner-Lange et al., 2020, Herfurth et al., 2022) and PilY1.3 was localized at the pilus tip via immunogold labeling (Treuner-Lange et al., 2020, Xue et al., 2022). Fluorescence microscopy revealed that localization of PilY1.3 and PilW3 to the polar T4aP machine (T4aPM) in *M. xanthus* is independent of the major pilin. PilW3 fails to localize in the absence of PilY1.3, and PilY1.3 localization is strongly dependent on PilV3 and PilW3, while PilX3 is required for the stabilization of PilY1.3 (Treuner-Lange et al., 2020). AlphaFold-Multimer modeling revealed a conserved order of proteins in the major pilin/minor pilin/PilY1 complexes 1 and 3 of *M. xanthus*: PilA (base) → FimU → PilW → PilV → PilX → PilY1 (tip), with the N-terminal domain of PilY1 forming the ultimate tip (Treuner-Lange et al., 2024). Interestingly, β-strand complementation between PilX and PilY1 proteins were predicted in both complexes, suggesting extraordinary stable protein interactions (Treuner-Lange et al., 2024). *In silico* analysis of PilY1 from the acidophilic bacterium *Acidithiobacillus thiooxidans* predicted specific residues in its C-terminal domain to be critical for interaction with the minor pilins PilW and PilX (Hernandez-Sanchez et al., 2024). Recently, deletion of minor pilins in *K. kingae* was shown to phenocopy the loss of PilC1 and PilC2, and a structural model of a complex composed of FimT, PilV, PilW, and PilX was successfully generated using AlphaFold (Yount et al., 2025a).

Cryo-ET showed *P. aeruginosa* PilY1 as a champagne-cork-shaped density in the outer membrane secretin pore (Guo et al., 2024), in contrast to the periplasmic cavity localization reported in *M. xanthus* (Treuner-Lange et al., 2020, Yount et al., 2025b). Future studies are needed to determine whether these localization differences reflect system-specific architecture or are conditional, as the *M. xanthus* study used cells grown in liquid (Treuner-Lange et al., 2020) while the *P. aeruginosa* study used cells grown on cryo- grids (Guo et al., 2024).

Latest emerging themes have connected mechanosensing properties of PilY1 proteins with structural plasticity. Single-molecule magnetic tweezers revealed that PilY1 from *P. aeruginosa* unfolds hierarchically, with mechanical stability increasing upon calcium binding, and that integrin binding via the RGD motif induces conformational changes, indicating force- and ligand-modulated function (Cao-Garcia et al., 2025).

Overall, the diverse localizations where PilY1 proteins are found in different bacteria include the cytoplasm, the inner membrane, the outer membrane, the length of the T4aP, the tip of T4aP, outer membrane vesicles, and the extracellular environment (Yount et al., 2025b), suggesting that PilY1 proteins also have functions outside the T4aP. Evidence for that comes from PilY1 from *P. aeruginosa*, which has been reported to function in processes such as secondary metabolism, cell density signaling, persistence, and virulence (Bohn et al., 2009, Pritchett et al., 2026). Increasing evidence also indicates that PilY1 is crucial for signaling upon surface contact in *P. aeruginosa* (Siryaporn et al., 2014, Luo et al., 2015, Persat et al., 2015). The model that the PilY1 protein, which is produced in the cytoplasm and transported to the periplasm, interacts with minor pilins to form a priming complex essential for T4aP assembly, which upon pilus elongation becomes a T4aP tip complex critical for adhesion and surface sensing is supported by data from a broad diversity of Gram-negative bacteria.

Nevertheless, some fundamental questions about the diversity and evolutionary background of PilY1 proteins remain unanswered, including the high variability of their N-terminal domains and how different PilY1 isoforms may have evolved to acquire distinct functional activities across lineages. In this context, the myxobacteria are of particular interest because this group includes bacteria that possess several PilY1 paralogs with different minor pilin complexes. These paralogs differ in their domains and expression levels, suggesting that PilY1 paralogs in myxobacteria may have evolved functional specializations in the T4aP machinery. However, the evolutionary background and genomic and structural diversification of PilY1 proteins in myxobacteria have not been explored so far. In this study, we systematically analyzed the diversity and classification of PilY1 isoforms across the phylum Myxococcota using comparative genomics, structural bioinformatics, and evolutionary approaches to elucidate their potential functional diversification and the versatility of the T4aP machinery.

## 2 Results

### 2.1 Comparative domain and motif analysis of well-studied PilY1 proteins from different organisms showcase the diversity amongst their domain architectures

**FIGURE 1:**
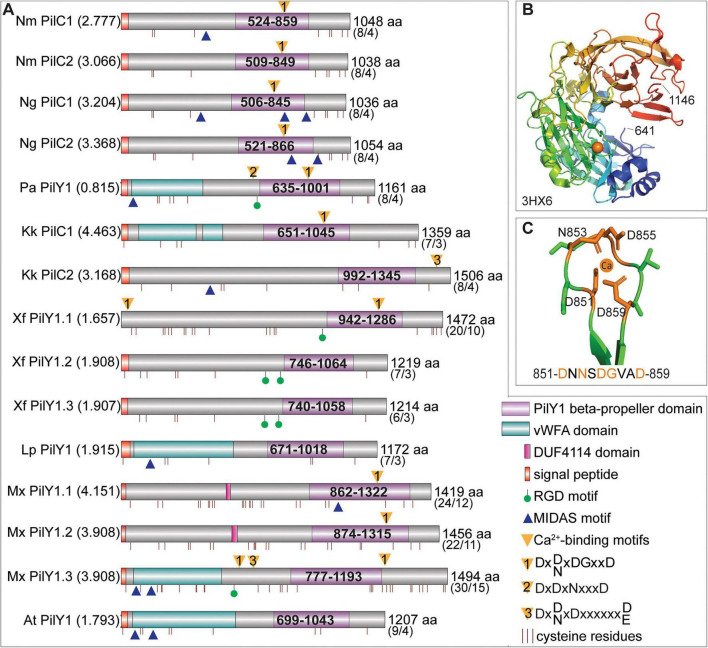
Domain structure and motifs of PilY1 proteins from various organisms. **(A)** Protein domains, motifs, and cysteine residues of selected PilY1 proteins are depicted and colored as explained in the figure legend in the bottom right corner. The depicted proteins are the as follows: Nm, *Neisseria meningitidis* FAM18 CAM09357 (PilC1), CAM09680 (PilC2); Ng, *N. gonorrhoeae* MS11 WP_353124200.1 (PilC1), WP_172763930.1 (PilC2); Pa, *Pseudomonas aeruginosa* PAO1 PA4554; Kk, *Kingella kingae* CRZ20459 (PilC1), CRZ19786 (PilC2); Xf, *Xylella fastidiosa* PD1611 (PilY1.1), PD0502 (PilY1.2), PD0023 (PilY1.3); Lp, *Legionella pneumophila* Q5ZXV3; Mx, *Myxococcus xanthus* MXAN_0362/_RS01780 (PilY1.1), MXAN_1020/_RS04905 (PilY1.2), MXAN_1365/_RS06610 (PilY1.3); At, *Acidithiobacillus thiooxidans* ATCC 19377 QFX95975. Protein length in amino acids (aa) is indicated at the end of each cartoon. Number of cysteines and predicted disulfide bridges in corresponding AlphaFold 3 models are shown in brackets below the length. RMSD values, obtained by superimposing corresponding AlphaFold model with the PDB structure 3HX6 (C-terminal part of the PilY1 protein from *P. aeruginosa*) are listed in brackets after the protein abbreviation. **(B)** Structure 3HX6 with the bound calcium molecule shown as an orange ball. **(C)** Structure, and sequence of the calcium-binding motif of the PilY1 protein from *P. aeruginosa.*

To explore the domain architecture diversity, we compiled the domain architectures of so far published PilY1/PilC proteins and compared the described domains and motifs, reported to be important for individual PilY1 function. While these proteins are all defined by their C-terminal beta-propeller domain, their lengths and N-terminal domains vary significantly (Figure 1A). A third of these proteins, coming from different bacterial phyla, have an N-terminal vWFA domain, while the DUF4114 domain is exclusively found in two myxobacterial PilY1 proteins (Figure 1A). Since the DUF4114 domain is found in many bacterial sugar-binding proteins, PilY1.1 and PilY1.2 were suggested to bind to EPS (Xue et al., 2022). Beside the N-terminal vWFA and DUF4114 domains, no other conserved sequences or structural domains have been identified to date (Figure 1A). Calcium-binding motifs (1, 2, and/or 3) (Cruz et al., 2014) and MIDAS motifs (Webster et al., 2022) are found in the majority of these PilY1 proteins (fifteen in numbers), whereas RGD motifs (Johnson et al., 2011) seem to be less common and are only found in a small subset of these proteins. The most common calcium-binding motif is motif 1 (Figure 1A), which was initially identified in the structure of the C-terminal domain of *P. aeruginosa* PilY1 (PDB ID: 3HX6, Figures 1B,C). Protein identities and similarities among the fifteen proteins are generally below 40% or 55%, respectively, whether the full-length proteins or only the beta- propeller domains are compared (Supplementary Table S1). The exceptions are the four *Neisseria* proteins and the two *X. fastidiosa* homologs, PilY1.2 and PilY1.3 (Supplementary Table S1). RMSD values calculated by superimposing the AlphaFold models of all fifteen PilY1 proteins onto the experimentally determined structure 3HX6 (Figure 1B) range from 0.8 to 4.5 Å, supporting the structural conservation of their β-propeller domains. In contrast, pairwise RMSD comparisons among the fifteen AlphaFold models in a one-to-one manner revealed only significant structural similarities between PilY1.1 and PilY1.2 of *M. xanthus* (RMSD < 3 Å), which do not correlate with sequence identity (Supplementary Table S1). This suggests that while the β-propeller fold is conserved, the overall structural architecture exhibits considerable variability across the different PilY1 proteins. Some PilY1 proteins are reported to form disulfide bridges (Cheng et al., 2013, Webster et al., 2022), and we observed that the number of cysteine residues in the PilY1 proteins varies from 7 to 30 residues with the three myxobacterial proteins having the highest numbers (Figure 1A). These differences are also reflected in the number of predicted disulfide bridges in the AlphaFold models (Figure 1A). Only three out of eight organisms encode a single PilY1 protein, while the other five organisms encode two or three different PilY1 proteins, indicating that bacterial genomes encoding more than one PilY1 protein are common (Figure 1A).

### 2.2 Three distinct minor pilin/PilY1 complexes shape T4aP tip architecture and function in *M. xanthus*

**FIGURE 2:**
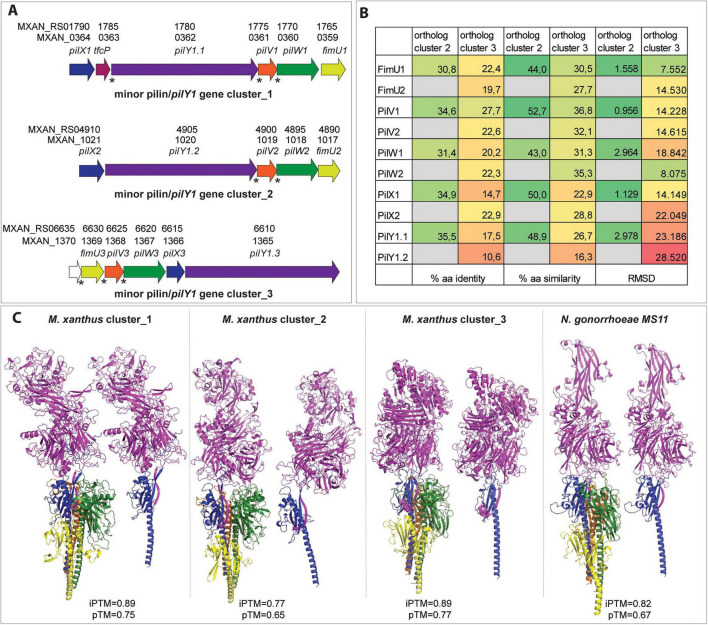
The three minor pilin/*pilY1* gene cluster of *M. xanthus* and the corresponding minor pilin/-PilY1 protein complexes. **(A)** Schematic representation of *M. xanthus* gene clusters encoding PilY1 (purple) and the four minor pilins FimU (yellow), PilV (orange), PilW (green) and PilX (blue). The *tfcP* gene in cluster 1 is shown in pink while *mxan_1370* is shown in white. The genes are shown with their old and new annotations, since the new annotation is used in the following sections. The star indicates translational coupling between the genes. **(B)** Amino acid identities (left) and similarities (middle) between proteins of cluster 1 and paralogs of cluster 2 and 3. RMSD values, obtained by superimposing corresponding AlphaFold models are shown at the right. Numbers are color-coded for clarity. **(C)** High-confidence AlphaFold models of the three minor pilin/PilY1 protein complexes from *M. xanthus* and *N. gonorrhoeae* MS11. The iPTM and pTM scores are shown below the models. FimU/T, PilV, PilW, PilX and PilY1 proteins are shown in the same colors as in A. For the model of the *N. gonorrhoeae* MS11 minor pilin/PilY1 (PilC) complex the following proteins were used for this model: WP_003690897.1_GspH/FimT, WP_003690898.1_PilV, WP_003690899.1_PilW, WP_003690900.1_PilX and WP_172763930.1_PilC. To ease visualization of the β-strand additions between PilX and PilY1 proteins, the models containing only PilX -PilY1 are also shown next to the complete models.

Three gene clusters (cluster 1–3) in *M. xanthus* encode each four minor pilins and a PilY1 protein (Treuner-Lange et al., 2020; Figure 2A). Cluster 1 further encodes an additional protein called TfcP, a non-canonical cytochrome c protein important for stabilizing PilY1.1 at low-calcium concentrations (Herfurth et al., 2022). Similarly, cluster three encodes an additional protein (MXAN_1370) (Figure 2A), which contains a T2SS-T3SS_pil_N Pfam domain but its function is not yet understood. The genes encoding TfcP and MXAN_1370 as well as some other genes in all three clusters are translationally coupled with up- or downstream genes (Treuner-Lange et al., 2020; Figure 2A), suggesting coordinated expression of these genes. Despite low sequence identity (<36%) and similarity (<53%) among the orthologs of the three clusters, AlphaFold models reveal significant structural conservation between PilY1 orthologs from cluster 1 and 2 (Figure 2B and Supplementary Table S1) and minor pilin orthologs (Figure 2B), indicating functional or evolutionary relatedness despite sequence divergence. Using AlphaFold modeling, high-confidence models of the minor pilin/PilY1 complexes from all three clusters were constructed, which revealed the same order of proteins, with FimU at the base and PilY1 at the top, with its N-terminal domain at the ultimate tip (Figure 2C). Since the conserved organization of *pilY1* and minor pilin encoding genes is found in several bacterial genomes (with exception of *Neisseria* sp.) (Winther-Larsen et al., 2005), we also modeled the minor pilin/PilY1 complexes from *N. gonorrhoeae* and found the same order of proteins in this complex (Figure 2C). In all four predicted minor pilin/PilY1 complexes, β-strand complementation between PilX and PilY1 proteins were predicted, as reported already for minor pilin/PilY1 complexes 1 and 3 of *M. xanthus* (Treuner-Lange et al., 2024). The presence of this extraordinary stable protein-interaction between PilX and PilY1 proteins conserved in all four complexes suggests that this feature might be of high functional relevance. Very recently, it was suggested that β-strand complementation might be a specific polymerization signal for any T4 filament system (Ellison et al., 2026).

The presence of three distinct minor pilin/*pilY1* gene clusters appears to enable *M. xanthus* to adapt its T4aP tip complexes to varying adhesion demands during social motility and potentially in response to environmental cues (Treuner-Lange et al., 2020, Xue et al., 2022, Kroos et al., 2025). The life cycle of *M. xanthus* comprises two main phases: (a) a vegetative phase, occurring in the presence of nutrients or prey, and (b) a developmental phase, which involves aggregation into fruiting bodies and sporulation under nutrient- or prey-limited conditions (recently reviewed in Kroos et al., 2025). EPS plays an essential role in T4aP-dependent motility, adhesion, biofilm formation and development of *M. xanthus* (Pérez-Burgos et al., 2020, Perez-Burgos and Søgaard-Andersen, 2020). RNA-seq analysis from a developmental course experiment shows that the minor pilin/*pilY1* clusters are differentially expressed, with cluster two being highly upregulated during development, while expression of clusters 1 and 3 occurs in the vegetative phase and during development (Supplementary Figure S1). Similar observations were made in other developmental time course experiments (Kuzmich et al., 2021, McLoon et al., 2021, Farrugia et al., 2025), and indicate that important regulators of development, such as FruA, CsgA and MrpC, are involved in expression of cluster 2 genes (McLoon et al., 2021, Farrugia et al., 2025). Further transcriptomic analyses reveal that in the vegetative phase the expression of clusters 1 and 3 is balanced by the transcription factor HsfA (Xue et al., 2022) and altered in mutants lacking EPS synthesis (Schwabe et al., 2026).

### 2.3 Major and minor pilin loci exhibit lineage-specific genomic organization

**FIGURE 3:**
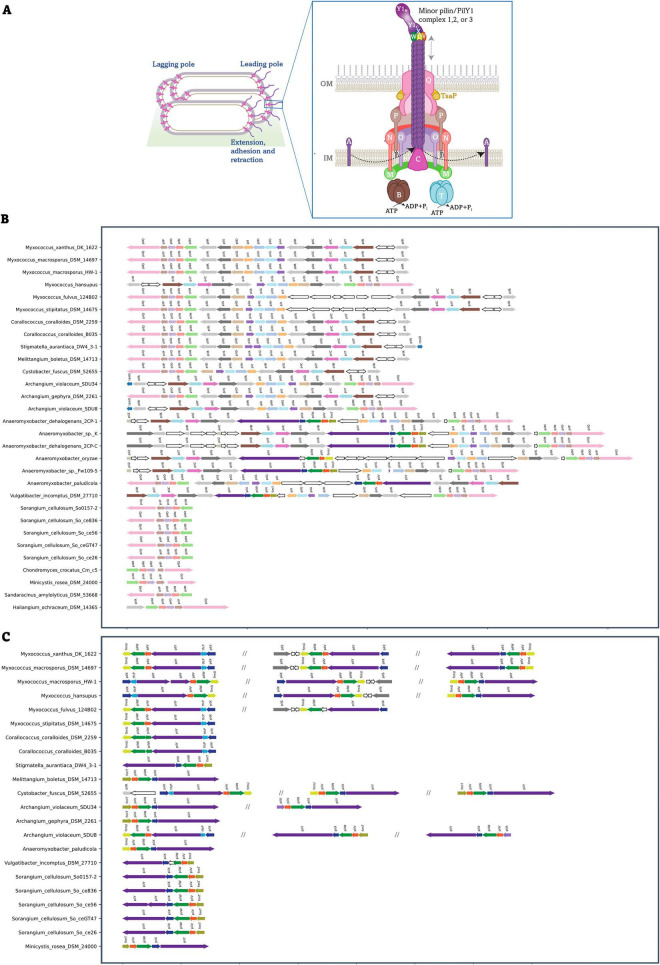
Genomic organization of major and minor pilin clusters in complete Myxococcota genomes. **(A)** Left: Cartoon of *M. xanthus* cells with unpiliated T4aP machine at the lagging cell pole and piliated T4aP machine at the leading cell pole. Right: The architecture of the T4aPM of *M. xanthus* in the piliated state is depicted with one minor pilin/PilY1complex tipping the T4a pilus. Other components of the piliated T4aPM are forming an OM pore (PilQ and TsaP), an alignment complex (PilP, PilN, and PilO) that connects the OM pore to the IM platform complex (PilC and PilM), and the ATPases PilB or PilT, which associate with PilM and PilC in a mutually exclusive manner for extension and retraction, respectively. Dashed bend arrows indicate incorporation at, and removal from, the pilus base of PilA during extension and retraction, respectively. Dashed arrows next to the T4a pilus indicate pilus extension and retraction. The proteins labeled with single letters have the Pil prefix, except for U, which corresponds to FimU. **(B)** Gene synteny of T4aP loci identified in complete genomes is shown in two panels. The panel **(B)** represents the major pilin clusters, while the panel **(C)** depicts the minor pilin clusters. Arrows indicate coding sequences, with orientation reflecting transcriptional direction. Homologous T4aP genes are color-coded consistently across genomes. Genes, identified as non-T4aP homologs, are represented by white arrows with black outlines. Although TsaP is depicted as part of the T4aP machinery, its genetic locus is not shown, as it is not associated with either the major pilin gene cluster or the minor pilin/PilY1 gene clusters.

In this study, the genomic organization, and loci of major and minor pilins in *M. xanthus* served as a reference for comparison across sixty-seven organisms in the phylum Myxococcota. Our comparative genomic analysis revealed that the major pilin gene cluster predominantly comprises of *pilQPONMRSDIHGARSCTB*, forming a single genomic locus that encodes nine core components of the T4aP machine (Figure 3A), supported by a previous study (Sharma et al., 2018a). In addition to this major *pil* cluster, this study showcases the diversity of minor pilin gene clusters, which each contain one *pilY1* gene and are typically found in synteny with *pilX*, *pilV*, *pilW*, and *fimU*. Notably, the cytochrome c gene, *tfcP*, known to present immediately upstream of *pilY1.1*, represents a conserved genomic organization at this locus for *pilY1.1* type cluster only (Figures 2A, 3 and Supplementary Figure S2).

Within the order Myxococcales, most genomes follow a genomic arrangement like that of *M. xanthus*, consisting of a major pilin cluster encoding the core structural genes and one to three *pilY1*-associated minor pilin clusters. However, members of the families Anaeromyxobacteraceae and Vulgatibacteraceae exhibit a distinct organization in which *pilY1*, *pilX*, *pilW*, *pilV*, and *fimU* are incorporated within the major pilin cluster itself. Among the six complete genomes of *Anaeromyxobacter* spp. examined, five displayed a single conserved genomic locus containing the pili genes. Notably, *Anaeromyxobacter paludicola* and *Vulgatibacter incomptus* DSM 27710 also encoded an additional *pilY1*-associated minor pilin cluster located downstream of the major pilin locus (Figure 3 and Supplementary Figure S2).

In the order Polyangiales, two principal pilin loci were typically observed: a cluster comprising *pilQPONM* and a separate *pilY1*-associated cluster. Exceptions to this pattern were identified in *Chondromyces crocatus* Cm c5 and *Sandaracinus amylolyticus* DSM 53668, which lacked a *pilY1* cluster. A similar absence of the *pilY1* cluster was observed in *Haliangium ochraceum* DSM 14365 of the order Haliangiales (Figure 3 and Supplementary Figure S2).

This study showcased a separate minor pilin cluster (Supplementary Figure S2), predominantly comprising homologs of *pilQ*, *pilB*, *pilC*, and *pilA*, in several genomes, however, further investigation revealed that this cluster belongs to type II secretion system (T2SS) (Konovalova et al., 2010). This cluster was comparatively larger in members of Polyangiales than in Myxococcales, with Polyangiales additionally encoding homologs of *pilE*, *pilW*, *pilM*, and *fimU* within the same genomic region (Supplementary Figure S2).

### 2.4 Pili-associated genes are broadly conserved and evenly distributed across Myxococcota

**FIGURE 4:**
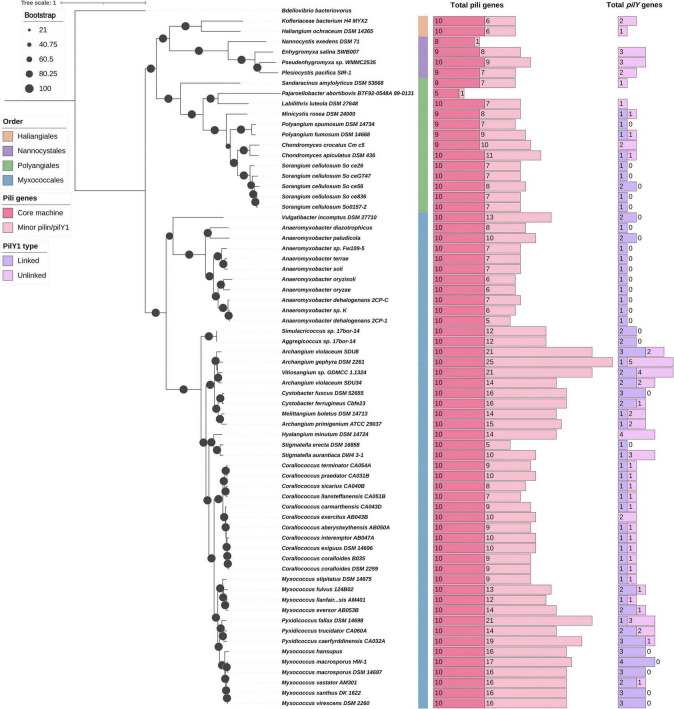
Maximum likelihood phylogeny of Myxococcota based on concatenated single copy orthologs showcasing the distribution of pili genes. Maximum likelihood (ML) phylogeny inferred using the LG + F + I + G4 model, identified as the best-fit substitution model according to BIC using ModelFinder (Kalyaanamoorthy et al., 2017) implemented in IQ-TREE v1.6.12 (Nguyen et al., 2015a). from a concatenated alignment of 237 single-copy orthologs comprising 120,768 sites. The tree is rooted with *Bdellovibrio bacteriovorus* (Kingdom Pseudomonadati, Phylum Bdellovibrionota). SH-aLRT and ultrafast bootstrap support values are shown above each branch. The scale bar represents the estimated number of substitutions per site. A color strip adjacent to the tree indicates the taxonomic order within the class. The first and second bar plots represent the total number of T4aP genes and the number of *pilY1* genes identified in each genome, respectively. The first stacked bar plot shows the counts of core and *minor pilin/pilY1* genes per organism. Core genes’ count includes one single copy of *pilQ*, *pilP, pilO, pilN, pilM, pilA, pilB, pilT, pilC*, and *tsaP*, while minor pilin/pilY1 genes’ count includes all copies of *pilV, pilW, pilX, pilY1*, and *fimU.* The second stacked bar plot depicts the distribution of linked and unlinked *pilY1* genes across genomes.

A comprehensive survey of the 67 genomes revealed that pili-associated genes are widely distributed across the phylum Myxococcota (Figure 4 and Supplementary Figure S3). This observation is consistent with a previous report based on a smaller dataset of 28 myxobacterial species (Sharma et al., 2018a) indicating that conservation of the T4aPM is a general feature of the phylum. When mapped onto the species phylogeny, members of the order Myxococcales encoded a slightly higher number of pili-associated genes compared to the other three orders. The lowest number of pili genes was detected in *Pajaroellobacter abortibovis*, an endosymbiont with a markedly reduced genome size of approximately 1.8 Mb relative to other myxobacteria (Figure 4). This reduction reflects genome streamlining associated with its parasitic and pathogenic lifestyle.

Core components of the T4aPM, including PilQ, PilP, PilO, PilN, PilM, PilA, PilC, PilT, PilB, and TsaP (Sharma et al., 2018a,Kroos et al., 2025; Figure 3A and Supplementary Figure S3), were consistently identified across the majority of genomes, indicating conservation of the structural backbone of the system, whereas variation was primarily observed among accessory components. Notable exceptions were observed for specific genes: *pilF* was not detected in members of Nannocystales and Haliangiales and was also absent in the families Anaeromyxobacteraceae and Vulgatibacteraceae within the order Myxococcales. In contrast, *pilE* was detected exclusively within Polyangiales. Similarly, *fimU* was not identified in Nannocystales and Haliangiales (Supplementary Figure S3).

Examination of PilY1 homologs across the dataset revealed their widespread presence, with PilY1 proteins broadly represented in most genomes analyzed. Consistent with previous reports, *M. xanthus* encodes three PilY1 proteins, designated PilY1.1, PilY1.2, and PilY1.3, all of which are present in linked clusters in synteny with *pilX*, *pilW*, *pilV*, and *fimU*. Across the 67 genomes examined here, the average copy number of PilY1 was two per genome. The highest copy number, six *pilY1* genes, was observed in *Archangium gephyra* DSM 2261 and *Vitiosangium* sp. GDMCC 1.1324, whereas *Pajaroellobacter abortibovis* and *Nannocystis exedens* DSM 71 did not encode any detectable PilY1 homolog (Figure 4 and Supplementary Table S2).

Genomic context analysis revealed variability in PilY1 organization. While several species, similar to *M. xanthus*, encoded PilY1 within linked clusters together with *pilX*, *pilW*, *pilV*, and *fimU*, other species exhibited *pilY1* in an unlinked configuration too, occurring as solitary genes outside canonical minor pilin operons. Many genomes contained a combination of both linked and unlinked *pilY1* copies. Within the order Myxococcales, members of the families Anaeromyxobacteraceae and Vulgatibacteraceae encoded exclusively linked PilY1, with *pilY1* positioned within the major pilin cluster in Anaeromyxobacteraceae, as described above. In contrast, members of Nannocystales and Haliangiales encoded only unlinked PilY1 (Supplementary Table S2). Although this pattern could partly reflect assembly fragmentation in draft genomes, the complete genome of *Haliangium ochraceum* DSM 14365 also encoded an unlinked PilY1, indicating that this organization is not solely attributable to genome incompleteness (Figure 4).

### 2.5 Phylogenetic analysis reveals distinct classes of PilY1 proteins across Myxococcota

**FIGURE 5:**
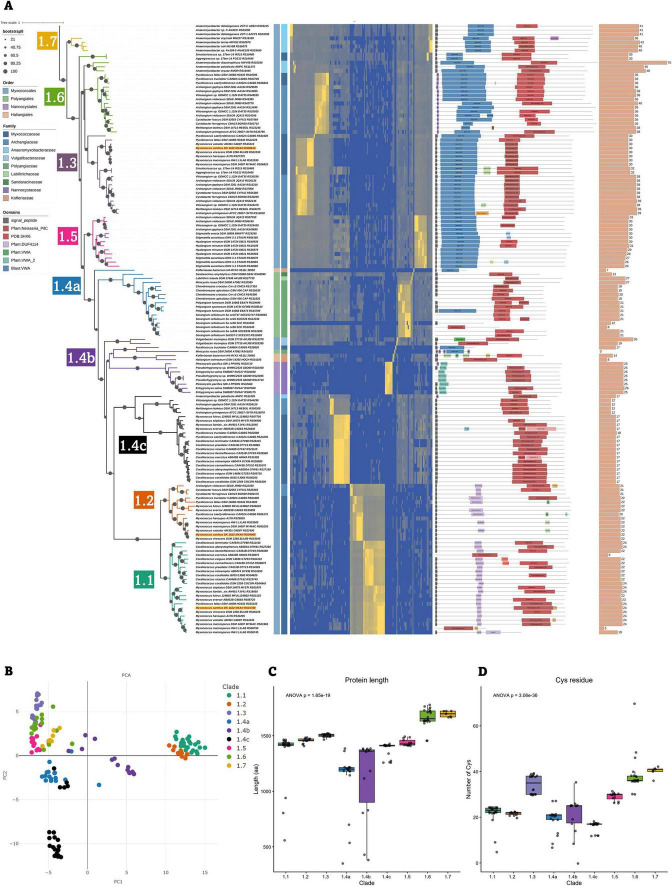
Phylogenetic characterization and classification of PilY1 proteins. **(A)** ML phylogeny of 150 PilY1 proteins inferred using the LG + F + I + G4 model with ultrafast bootstrap support values are shown above each branch. The initial multiple sequence alignment comprised 4,031 sites and was trimmed using trimAl v1.5 in gappyout mode (Capella-Gutiérrez et al., 2009) to 337 informative positions prior to tree construction. Clades are color-coded according to the classes identified. The first and second annotation strips indicate order-level and family-level taxonomy, respectively. Conserved domains detected using CDD are mapped onto each sequence according to their positional coordinates along the protein length. The terminal bar plot represents the total number of cysteine residues in each protein. **(B)** Principal component analysis (PCA) of PilY1 proteins based on percentage identity across members, showing clustering consistent with the phylogenetic clade assignments. **(C)** Boxplot showing the distribution of protein length (amino acid residues) of PilY1 proteins grouped by clade. **(D)** Boxplot showing the distribution of cysteine residue counts across PilY1 proteins grouped by clade.

Phylogenetic analysis of PilY1 proteins revealed the presence of distinct clades that could be segregated based on sequence similarity (percentage identity), domain architecture, protein length, and the number of conserved cysteine residues. These clades could be clearly demarcated in the PilY1 phylogeny, indicating substantial divergence among PilY1 homologs across Myxococcota (Figure 5). *M. xanthus* PilY1.1 and PilY1.2 are similar to each other (Figures 1A, 2B), following which, the clades containing *M. xanthus* PilY1.1 and PilY1.2 also formed sister groups in this phylogeny and were distinct from other clades in their domain architecture. These two clades also uniquely contained the DUF4114 domain, which was absent from all other PilY1 clades. Although the DUF4114 domain was not detected in all members under stringent cutoff thresholds using CDD (Figure 5A and Supplementary Table S3), alignment of the corresponding DUF4114 region revealed a high degree of residue conservation across all proteins within clades 1.1 and 1.2, supporting the presence of this domain in these groups (Supplementary Figure S4). Members of these clades were restricted to the order Myxococcales (Figure 5A). The median number of cysteine residues is found to be very similar in these two clades, with 23 in clade 1.1 and 22 in clade 1.2. Within clade 1.1, two annotated proteins from *M. macrosporus* HW-1, LILAB_RS06745 and LILAB_RS06750, were predicted to contain the beta-propeller domain and the DUF4114 domain, respectively. However, the separation of these domains into two adjacent proteins likely reflects a gene annotation error. Domain architecture and genomic context suggest that these entries represent fragments of a single full-length PilY1 protein containing both the beta-propeller and DUF4114 domains (Figures 3, 5A).

PilY1.3 of *M. xanthus* forms a separate and phylogenetically distant clade from clades 1.1 and 1.2. Members of this clade encode a vWFA domain at the N-terminus and, similar to clades 1.1 and 1.2, are restricted to the order Myxococcales. This clade exhibited a higher median cysteine content of 35 residues, which is significantly higher than 22/23 for PilY1.1 and PilY1.2. Beyond the three clades containing the *M. xanthus* PilY1 paralogs, six additional clades were identified: 1.4a, 1.4b, 1.4c, 1.5, 1.6, and 1.7 (Figure 5A).

Clade 1.4 forms the sister lineage to clades 1.1 and 1.2, with clade 1.4c representing the immediate sister group. Proteins in the clade 1.4c contain only a beta-propeller domain toward the C-terminus and are also confined to Myxococcales. This clade displayed the lowest median cysteine content, with seventeen residues (Figures 5A,D). In contrast, clade 1.4b is the only clade comprising members from all four orders and showed a median cysteine content of twenty-five residues. These proteins encode both a vWFA domain and a beta-propeller domain; however, the vWFA domain is shorter than that observed in clade 1.3 proteins (Figure 5A). Notably, multiple paralogs from the same organism cluster together within this clade. For example, two proteins from *V. incomptus* DSM 27710, AKJ08_RS03395 and AKJ08_RS10270, group within this lineage, where AKJ08_RS03395 is associated with the major pilus cluster and AKJ08_RS10270 corresponds to the minor pilin locus (Figures 3, 5A and Supplementary Figure S2). Additionally, three PilY1 proteins from *Pseudenhygromyxa* sp. WMMC2535 and *Enhygromyxa salina* SWB007 fall within clade 1.4b; however, these represent unlinked *pilY1* genes (Figure 4). Within these two organisms in two instances, the *pilY1* gene is syntenic with *pilX*, whereas in the third case, *pilX* and *pilW* are located in the immediate genomic neighborhood. Importantly, all three *pilY1* genes of *P. sp.* WMMC2535 are located on the same contig, and none are positioned at contig edges, supporting the conclusion that their unlinked organization reflects a genuine triplicated genomic arrangement rather than an artifact of incomplete assembly. Clade 1.4b and 1.4c mostly include unlinked PilY1 proteins of the dataset (Supplementary Table S3).

Clade 1.4a comprises members of the order Polyangiales and represents the only clade lacking any representatives from Myxococcales. Almost all linked and unlinked PilY1 proteins from Polyangiales cluster within this lineage, with the sole exception of A7982_RS41025 from *Minicystis rosea* DSM 24000. Proteins belonging to clade 1.4a are the shortest among all PilY1 sequences in the dataset and have a median cysteine content of twenty-one residues (Figure 5D). Additionally, a gene annotation inconsistency similar to that observed in *M. macrosporus* HW-1 was identified in *Sorangium cellulosum* So ce56, where SCE_RS10345 and SCE_RS10350 appear to represent fragments of a single PilY1 protein that were incorrectly annotated as two separate coding sequences (Figures 3, 5A).

Clades 1.5, 1.6, and 1.7 display domain architectures highly similar to that of clade 1.3, and all three clades include members of the order Myxococcales. The median cysteine counts increase progressively across these clades, with thirty residues in clade 1.5, 36 in clade 1.6, and forty-one in clade 1.7, the latter representing the highest median cysteine content observed in the dataset (Figures 5A,D). All orthologs from the genera *Hyalangium* and *Stigmatella* cluster within clade 1.5. Proteins belonging to clades 1.6 and 1.7 are the longest among all PilY1 sequences in the dataset, and clade 1.7 includes members of the genus *Anaeromyxobacter*, further highlighting lineage-specific diversification within these extended and cysteine-rich PilY1 variants.

Consistent with these observations, principal component analysis further supported this clade-level classification, with members of each clade forming distinct clusters (Figure 5B). Moreover, comparative plots of protein length (Figure 5C) and cysteine residue distribution (Figure 5D) demonstrated statistically significant differences among clades, underscoring that both size variation and cysteine enrichment contribute to the structural and evolutionary differentiation of PilY proteins across the dataset (Figure 5).

**FIGURE 6:**
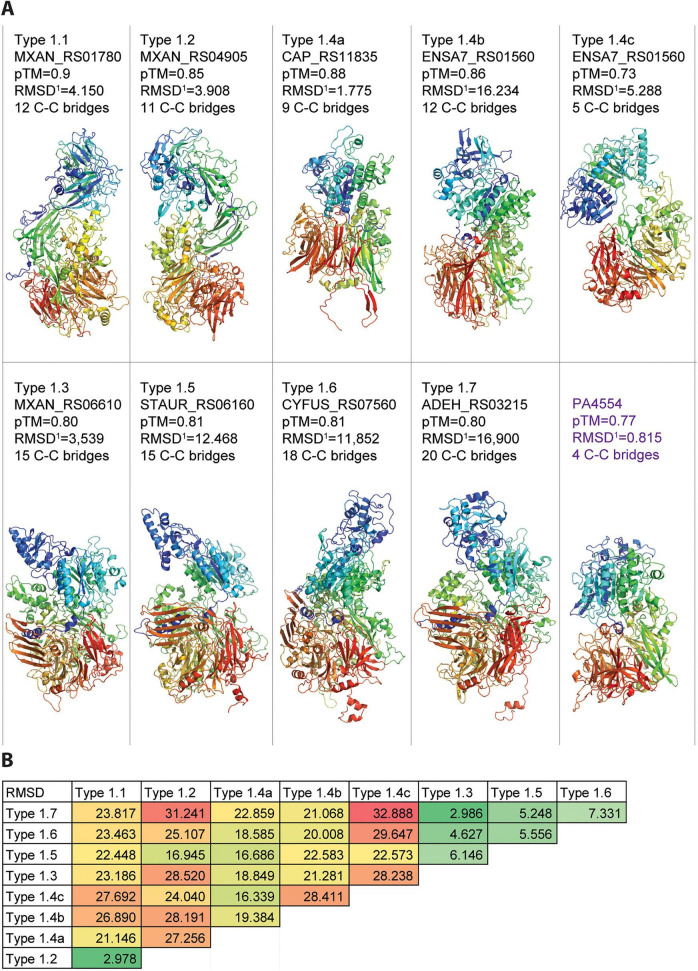
AlphaFold models of myxobacterial PilY1 proteins of each clade. **(A)** AlphaFold models of the indicated PilY1 proteins are displayed in rainbow coloring, ranging from blue at the N-terminus to red at the C-terminus. The pTM scores for each model are shown below the corresponding locus tags. RMSD values (RMSD^1^), obtained by superimposing each AlphaFold model with the PDB structure 3HX6 (C-terminal domain of *P. aeruginosa* PilY1), are listed beneath the pTM scores. The predicted number of disulfide bridges for each model is indicated below. **(B)** RMSD values obtained by pairwise superposition of the indicated myxobacterial PilY1 AlphaFold models are shown, with numbers color-coded for clarity.

These similarities are also reflected when comparing the RMSD measurements retrieved by superimposing AlphaFold models of example PilY1 proteins of all nine types (Figure 6). RMSD values below 3 are only obtained between *M. xanthus* PilY1.1 and PilY1.2 and *M. xanthus* PilY1.3 and the PilY1.7 type PilY1 protein from *A. dehalogenans* (Figure 6). Moderate RMSD values are found between the example proteins of the types PilY1.5, 1.6 and 1.7 with PilY1.3 (Figure 6). All other comparisons indicate significant structural differences. The high sequence and structural similarity between *M. xanthus* PilY1.1 and PilY1.2 suggest that the DUF4114 domain is embedded within a larger region exhibiting conserved architecture. This was confirmed by superimposing the AlphaFold models of the two proteins lacking the β-propeller and C-terminal domains (Supplementary Figure 5A). The RMSD value decreased to 1.982, indicating significant structural conservation in the N-terminal region. This similarity is visualized in the overlay model shown in Supplementary Figure 5A. As illustrated, the DUF4114 domain is part of a larger structural unit composed of multiple β-strands forming β-sheets, which are interrupted by several α-helices (Supplementary Figure 5A). Foldseek searches using *M. xanthus* PilY1.1 identified structurally similar proteins primarily from the PilY1/DUF4114 family across diverse bacterial lineages. Among the top hits is a PilY1 protein from *Desulfonema ishimotonii* (Di PilY1), which also contains an N-terminal DUF4114 domain (Supplementary Figure 5A). The moderate structural similarity between the N-terminal regions of *M. xanthus* PilY1.1 and Di PilY1 RMSD of 8.441 is shown in an overlay model (Supplementary Figure 5A). These findings clearly demonstrate that PilY1 proteins containing a DUF4114 domain are not restricted to the phylum *Myxococcota* but are evolutionarily also conserved in Thermodesulfobacteriota and likely across distantly related bacterial lineages. We also saw high variations in the number of predicted disulfide bridges in these models reaching from 5 to 20 within an individual PilY1 model, whereas the model of the *P. aeruginosa* PilY1 protein predicted only four disulfide bridges (Figure 6).

The distribution of conserved motifs across PilY1 proteins varied among the identified clades. Members of clades 1.1 and 1.2 predominantly contained the Ca^2+^-binding motif 1, whereas most proteins belonging to clades 1.4c, 1.4a, and 1.7 lacked all three predicted Ca^2+^-binding motifs. Across the entire dataset of 150 PilY1 proteins, only five sequences were found to contain all three Ca^2+^-binding motifs simultaneously. In contrast, the presence or absence of the MIDAS motif did not show clear clade-specific confinement and was distributed across multiple lineages. The RGD motif was detected in twenty-nine of the 150 PilY1 proteins, indicating that this motif is present only in a subset of PilY1 homologs within Myxococcota (Supplementary Table S3).

### 2.6 NR phylogeny supports vertical inheritance of PilY1.1 and PilY1.2 while the remaining PilY1 classes show patterns consistent with horizontal acquisition

**FIGURE 7:**
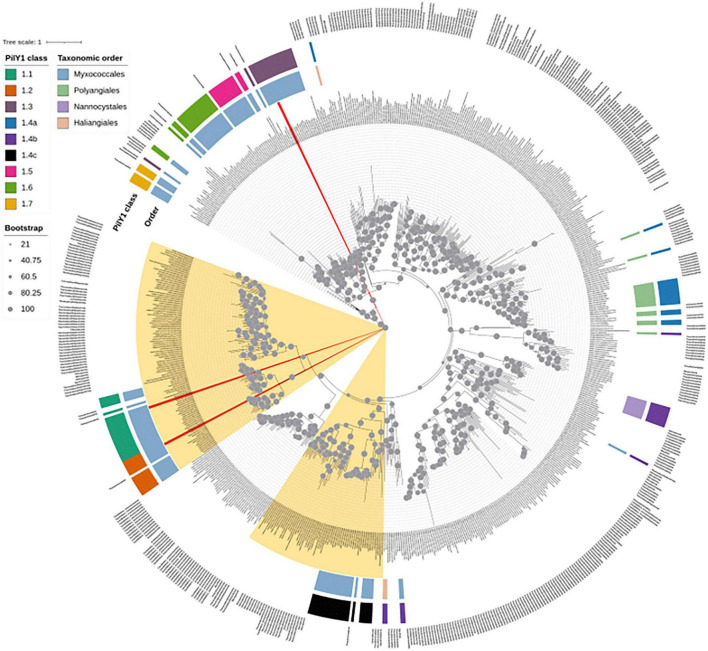
Phylogenetic placement of PilY1 homologs in the NR database. ML phylogeny constructed using the top twenty-five homologs for each of the 150 identified PilY1 proteins retrieved from the NR database. The tree was inferred under the WAG + G4 substitution model, and ultrafast bootstrap support values are shown above each branch. Branches corresponding to *M. xanthus* PilY1.1, PilY1.2, and PilY1.3 are highlighted in red. Clades containing homologs from Thermodesulfobacteriota are highlighted in yellow. The first annotation strip indicates the taxonomic order of the members in our dataset, the second strip represents the different PilY1 classes, and the third strip denotes the phylum-level affiliation of the corresponding NR homolog.

To investigate the evolutionary origin of the PilY1 paralogs detected in this study, homologs of each protein were retrieved from the NR database and subjected to phylogenetic analysis. Within the NR phylogeny, all detected classes of PilY1 formed distinct clades. PilY1.1 and PilY1.2 formed a well-supported sister clade, with their closest neighboring sequences belonging predominantly to the phylum Thermodesulfobacteriota, previously classified within the Deltaproteobacteria. Members of the clade 1.4c displayed a similar phylogenetic affinity, grouping nearest to homologs from Thermodesulfobacteriota (Figure 7). This shared phylogenetic proximity, together with their restricted distribution within Myxococcales in our dataset, supports the hypothesis that these paralogs were inherited vertically.

In contrast, PilY1.3 formed a distinct lineage with PilY1.5 and PilY1.6 as the sister clades. These clades included homologs from diverse phyla, including Pseudomonadota, Actinomycetota, Chloroflexota, and Gemmatimonadota. The broad taxonomic distribution and phylogenetic positioning of this lineage suggest that PilY1.3, PilY1.5 and PilY1.6 may have been acquired through horizontal gene transfer, followed by subsequent diversification within Myxococcales. Similarly, clades 1.4a and 1.4b showed the closest phylogenetic affinity to homologs from the phylum Pseudomonadota (Figure 7).

## 3 Discussion

This comprehensive genomic, structural, and phylogenetic study reveals that PilY1 proteins in Myxococcota have undergone extensive diversification, resulting in multiple well-resolved clades that differ in domain architecture, protein length, cysteine content, and genomic organization. The concordance between phylogenetic construction, domain composition, synteny patterns, and principal component analysis supports the robustness of the classification and indicates that PilY1 evolution in this lineage has been shaped by both vertical inheritance and horizontal gene transfer.

The shared ancestry between *M. xanthus* PilY1.1 and PilY1.2 suggests that these paralogs likely arose from a gene duplication event, a process that is highly prevalent in myxobacteria (Goldman et al., 2006). Their restriction to Myxococcales and close phylogenetic proximity to Thermodesulfobacteriota members further support vertical inheritance. Their shared and highly conserved DUF4114 domain, despite not always being detected under stringent CDD thresholds, further reinforces their common ancestry. The high residue conservation across the DUF4114 region suggests functional constraint and likely conservation of structural or interaction properties specific to these paralogs. Together, these observations indicate that PilY1.1 and PilY1.2 represent lineage-specific duplications that were retained and functionally maintained within Myxococcales.

In contrast, PilY1.3 displays a markedly different evolutionary trajectory. Its placement in a phylogenetically distant clade containing homologs from Pseudomonadota, Actinomycetota, Chloroflexota, and Gemmatimonadota suggests a history of horizontal acquisition, consistent with the prevalence of horizontal gene transfer events reported in myxobacteria (Sharma et al., 2018b,Chambers et al., 2020). The broader taxonomic distribution of this clade, combined with its distinct domain architecture characterized by an N-terminal vWFA domain and significantly higher cysteine content, supports the hypothesis that PilY1.3 originated from a non-Myxococcales lineage and was subsequently integrated and diversified within Myxococcales. This horizontal acquisition may have expanded the functional repertoire of the T4aP system in these soil social-motile bacteria.

The remaining PilY clades further illustrate the dynamic evolution of this protein family. Clade 1.4a, composed almost exclusively of Polyangiales members and lacking Myxococcales representatives, represents a distinct lineage characterized by reduced protein length and comparatively lower cysteine content. The confinement of nearly all Polyangiales PilY1 homologs to this clade suggests either lineage-specific specialization or differential selective pressures shaping pilus-associated functions in this order. The absence of Myxococcales sequences in clade 1.4a underscores the deep divergence between these orders within Myxococcota.

Clade 1.4b is notable for its broad taxonomic distribution across all four orders and for harboring both linked and unlinked pilY1 genes. The presence of multiple paralogs from the same organism within this clade, along with conserved syntenic associations with *pilX* and *pilW*, suggests ongoing duplication and functional partitioning. Importantly, the observation that unlinked *pilY1* genes in *P. sp.* WMMC2535 are located on the same contig and away from contig edges confirms that their dispersed organization reflects genuine genomic architecture rather than assembly artifacts. Such genomic dispersion may indicate modular recruitment of PilY1 variants into different pilus contexts.

Clades 1.5, 1.6, and 1.7 represent another axis of divergence, characterized by increased protein length and progressive enrichment of cysteine residues. The median cysteine count rises from clade 1.5 to 1.7, with clade 1.7 displaying the highest values in the dataset. Proteins in clades 1.6 and 1.7 are also the longest PilY1 variants identified. Elevated cysteine content may reflect increased disulfide bond formation, potentially contributing to structural stabilization of extended extracellular domains. The confinement of specific genera, such as *Hyalangium*, *Stigmatella*, and *Anaeromyxobacter*, to particular clades further indicates lineage-specific expansions and adaptation.

Across all clades, statistically significant differences in protein length and cysteine content demonstrate that these are not arbitrary phylogenetic subdivisions but represent structurally and biochemically distinct groups. Increased cysteine content may promote disulfide bond formation within extracellular domains, potentially enhancing structural stability of these large adhesins in the periplasmic or extracellular environment. Principal component analysis based on these parameters recapitulated the phylogenetic clustering, with each clade forming discrete groups in multivariate space. This convergence of phylogenetic, structural, and quantitative analyses underscores the evolutionary depth of PilY1 diversification in Myxococcota. Taken together, our findings suggest a model in which the ancestral Myxococcota PilY1 repertoire underwent lineage-specific duplications, domain rearrangements, and occasional horizontal acquisition events. Vertical inheritance appears to dominate core paralogs such as PilY1.1 and PilY1.2, whereas PilY1.3 and related variants likely represents a horizontally acquired innovation. Subsequent expansion and divergence across orders and genera have generated a structurally diverse family of PilY1 proteins that may contribute to functional specialization of the T4aP system in different ecological and developmental contexts.

Importantly, our structural modeling of minor pilin-PilY1 complexes indicates that the overall architecture of the priming complex remains conserved, with PilY1 positioned at the distal tip of the assembly. The predicted β-strand complementation between PilX and PilY1 proteins across several modeled complexes suggests a conserved interaction mechanism that may stabilize the tip complex during both pilus extension and retraction. This structural conservation, despite substantial sequence diversity in the N-terminal regions, supports a model in which the PilY1 C-terminal domain aims to maintain the structural integrity of the T4aP complex while the N-terminal domain helps in diversifying to enable functional specialization in adhesion or environmental sensing.

In the context of *M. xanthus*, the presence of three distinct minor pilin-PilY1 complexes suggests an adaptive strategy that may allow the bacterium to deploy different T4aP tip structures depending on environmental or developmental conditions. Transcriptomic analyses support this hypothesis by revealing differential expressions of the three minor pilin/*pilY1* clusters during vegetative growth and development. Although PilY1.1 and PilY1.3 have been detected in purified sheared pilus fractions (Treuner-Lange et al., 2020, Herfurth et al., 2022) and only PilY1.3 was found at pilus tips by immunogold (Treuner-Lange et al., 2020, Xue et al., 2022), it is tempting to speculate that PilY1.1 and PilY1.2 also cap the T4a pilus via their N-terminal DUF4114 domains, which are implicated in EPS binding. Based on our current model, T4aP-dependent motility and adhesion are mediated by phase-specific interactions of PilY1 proteins: PilY1.1 binds EPS during growth and development, PilY1.2 engages EPS during late development, and PilY1.3 may interact with both biotic and abiotic surfaces across all stages. This model is supported by the observation that PilY1.3 enables T4aP-dependent motility in the absence of EPS (Xue et al., 2022), suggesting a functional redundancy or adaptability in surface sensing. This mechanism likely enhances the environmental versatility of the *M. xanthus* T4aP system. Notably, a similar principle may apply to the Kil (Tad) pilus of *M. xanthus*, where four distinct minor pilin complexes are proposed to form interchangeable tips, enabling interactions with diverse prey cells (Herrou et al., 2025).

## 4 Material and methods

### 4.1 Domain and motif searches

Domain cartoons as depicted in Figure 1 are based on manual searches for calcium-binding motifs, MIDAS- and RGD-motifs using the SnapGene program (Version 8.2.1).^1^ For signal peptide predictions SignalP-6.0 was used (Teufel et al., 2022), predictions of the vWFA domain were done using SMART (smart.embl.de), PilY1 beta-propeller and DUF4114 domains were predicted using CD-Search (NCBI) against the Pfam database with *e*-values of 0.1. Amino acid motifs such as RGD, MIDAS (DxSxS), and calcium-binding motifs 1–3 as well as the number and position of cysteines residues were manually searched using SnapGene (Version 8.2.1). Domains and motifs were illustrated using DOG2.0.

### 4.2 Analysis of sequence identity and similarity

To calculate amino acid sequence identities and similarities the EMBOSS needle program (version 6.6.0) was used (Madeira et al., 2024).

### 4.3 Structural predictions and comparisons

AlphaFold 3 was used for structure prediction of minor pilin/PilY1 complexes as well as PilY1 proteins (Abramson et al., 2024). Structural differences between protein conformations were quantified using PyMOL v2.5.7 (Schrödinger LLC) via one-to-one Root Mean Square Deviation (RMSD, in Å) calculations, with visualization performed using the same software. If not otherwise stated, AlphaFold 3 models of full-length proteins were compared. Foldseek (van Kempen et al., 2024) was used to screen for proteins with structural similarities.

### 4.4 Dataset selection

A dataset of sixty-seven myxobacterial species was curated for this study, and the corresponding genomic and proteomic data were downloaded from the NCBI database (Sayers et al., 2025). Species were selected to ensure representation across all currently described families within the phylum Myxococcota. The final dataset included thirty-three complete genomes and thirty-four draft genome assemblies. Both assembly types were retained to maximize taxonomic breadth while maintaining sufficient genomic resolution for downstream identification and comparative analyses of pili-associated proteins.

### 4.5 Detection of proteins of interest

The proteomes of the selected genomes were subjected to both homology-based and profile-based approaches to identify genes associated with T4P motility and related systems. Homology-based searches were performed using BLAST v2.13.0 (Altschul et al., 1990) with an e-value cutoff of 10^−5^ against curated reference sequences of known pili proteins encoded by *M. xanthus*. Hits were filtered based on sequence similarity, alignment coverage, and statistical significance to retain only the high confidence candidates. For profile based detection, hidden Markov model (HMM) profiles were constructed for proteins associated with the pilus system, including those involved in twitching motility, chemosensory pili pathways, positive phototactic motility, pilus assembly, and fimbrial functions, based on orthology assignments obtained from the KEGG database (Brite: ko02035) (Kanehisa and Goto, 2000). These curated profiles were compiled into a custom database of fifty-seven profiles of the pilus system. Each proteome was scanned against this database using hmmscan of HMMER v3.4 suite (Eddy, 2011) with an e-value cutoff of 10^−5^. In addition, the TsaP gene was identified using an iterative search strategy using jackhmmer implemented in the HMMER v3.4 suite (Eddy, 2011), taking the TsaP sequence from *P. aeruginosa* as the query under the same threshold.

In parallel, searches were also conducted against the Pfam database (Mistry et al., 2021) to identify conserved domains associated with detected pili proteins. To complement these approaches, MacSyFinder v2.1.5 (Néron et al., 2023) with default parameters was also employed using appropriate models for pili machinery detection. For PilY proteins, domain architecture was examined in detail and the conserved domains were identified using hmmscan against the Conserved Domain Database (Wang et al., 2023) with an e-value cutoff of 10^−5^, allowing characterization and classification of functional modules encoded within PilY1 homologs across the dataset.

### 4.6 Phylogenetic inference

For phylogenetic analyses, both single copy orthologs and selected gene specific datasets were examined. Single copy orthologs across the sixty-seven genomes were identified using Proteinortho v6.3.1 (Klemm et al., 2023) with default parameters. The resulting orthologous groups were aligned individually using MAFFT v7.526 (Katoh and Standley, 2013) with the auto option. The alignments of single copy orthologs were concatenated to generate a species level phylogeny. In addition, phylogenetic trees were constructed for PilY1 proteins detected within the dataset to examine their evolutionary relationships. To place these sequences in a broader evolutionary context, PilY1 homologs were identified by searching against the NCBI non-redundant database (Sayers et al., 2025) downloaded on 14 April 2025 using DIAMOND v2.1.0.154 (Buchfink et al., 2021) with an e-value cutoff of 10^−5^. Homologous sequences retrieved from the database were incorporated into subsequent alignments and phylogenetic analyses. Maximum likelihood trees were reconstructed using IQ-TREE v1.6.12 (Nguyen et al., 2015a) and were visualized and annotated using iTOL v6 (Letunic and Bork, 2024).

### 4.7 RNA-seq analysis

RNA sequencing data were obtained from the study on gene expression during developmental cycle in *M. xanthus* (Sharma et al., 2021). The dataset was reanalyzed to examine the expression patterns of genes of interest identified in the present study, particularly those associated with the pilus motility and related sensing systems. Expression values corresponding to the selected genes were extracted and compared across the relevant experimental conditions described in the original study.

### 4.8 Statistical analysis and visualization

Statistical analyses and data visualization were performed using R and Python. In R, packages including ggplot2, ggpubr, dplyr, and the tidyverse framework were used for data visualization, statistical testing, and figure generation. In Python, analyses and plotting were conducted using pandas and matplotlib.

## Data Availability

The original contributions presented in the study are included in the article/Supplementary material, further inquiries can be directed to the corresponding authors.

## Footnotes

1
www.snapgene.com
