# Supplementary material for: Diversity, classification, and evolution of myxobacterial PilY1 proteins
Source: Front Microbiol. 2026 May 7;17:1826482. doi: 10.3389/fmicb.2026.1826482 (PMC13190605; doi:10.3389/fmicb.2026.1826482)
Supplement: Supplementary file 1 [file Data_Sheet_1.pdf]

Supplementary Material

Diversity, classification, and evolution of myxobacterial PilY1 proteins

Utkarsha Mahanta[1], Roman Waßmuth[2], Sherin Brighty[1], Anke Treuner-Lange[2*,] Gaurav Sharma[1*]

[1] Department of Biotechnology, Indian Institute of Technology Hyderabad, Sangareddy, Telangana, India 502284

[2] Max Planck Institute for Terrestrial Microbiology, 35043 Marburg, Germany

*** Correspondence:**
Corresponding Authors
Anke.Treunerlange@mpi-marburg.mpg.de; sharmag@bt.iith.ac.in

1 **Supplementary Data**

This PDF file includes

Supplementary Figures 1-5

Other supplementary material for this manuscript includes the following:

Supplementary Tables 1-3

Supplementary Figures:

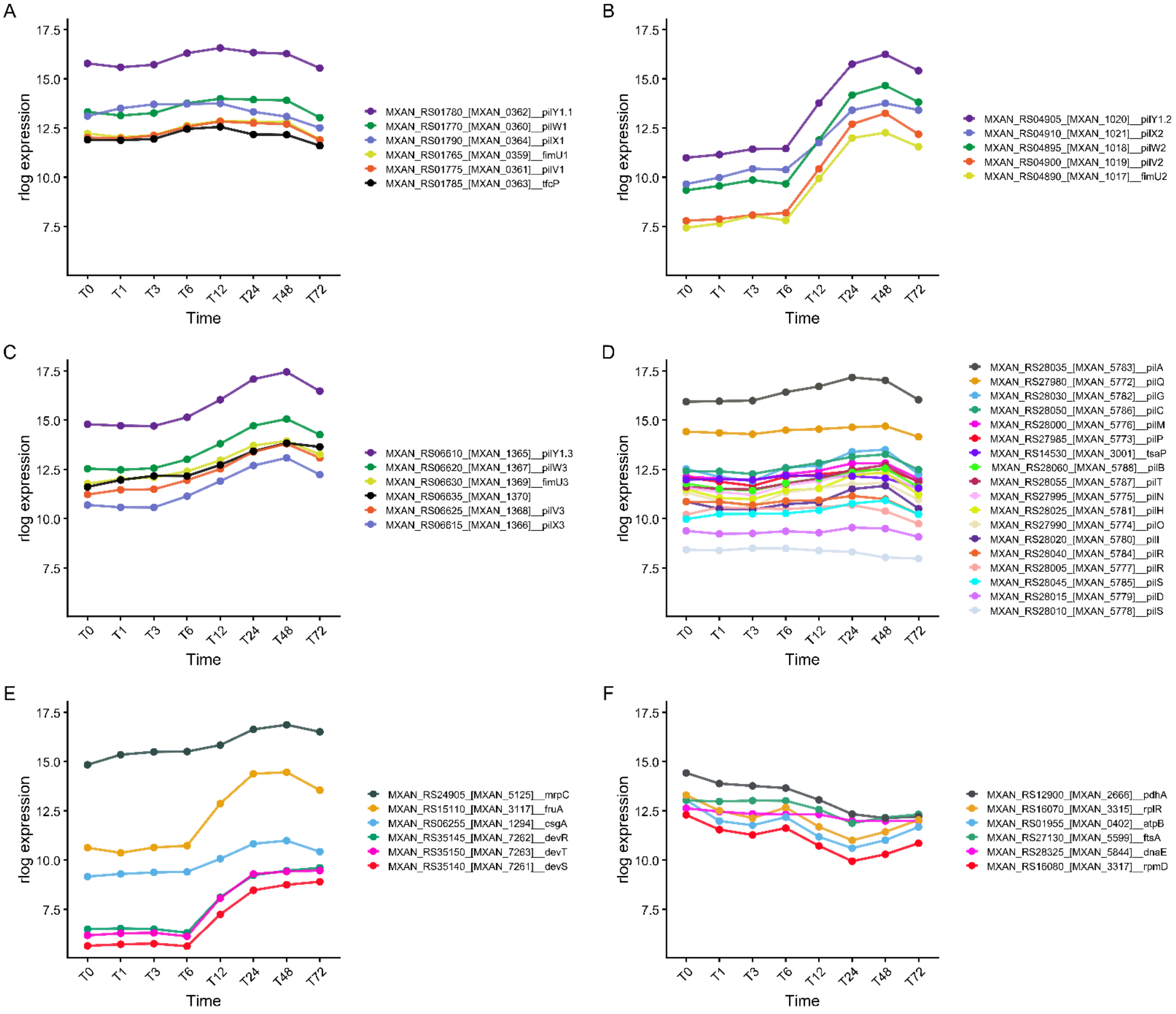

Supplementary Figure S1: RNA-seq expression profiles of minor and major pilin gene clusters in *Myxococcus xanthus*. RNA-seq–derived expression profiles of three minor pilin gene clusters (A-C) and the major pilin gene cluster (D) are presented in the upper and middle panels. The lower panel represents controls, showing the expression patterns of genes known to be upregulated during the developmental (E) and vegetative (F) stages in *M. xanthus*. Genes are labeled with locus tags of old and new genome annotations as well as their trivial names.

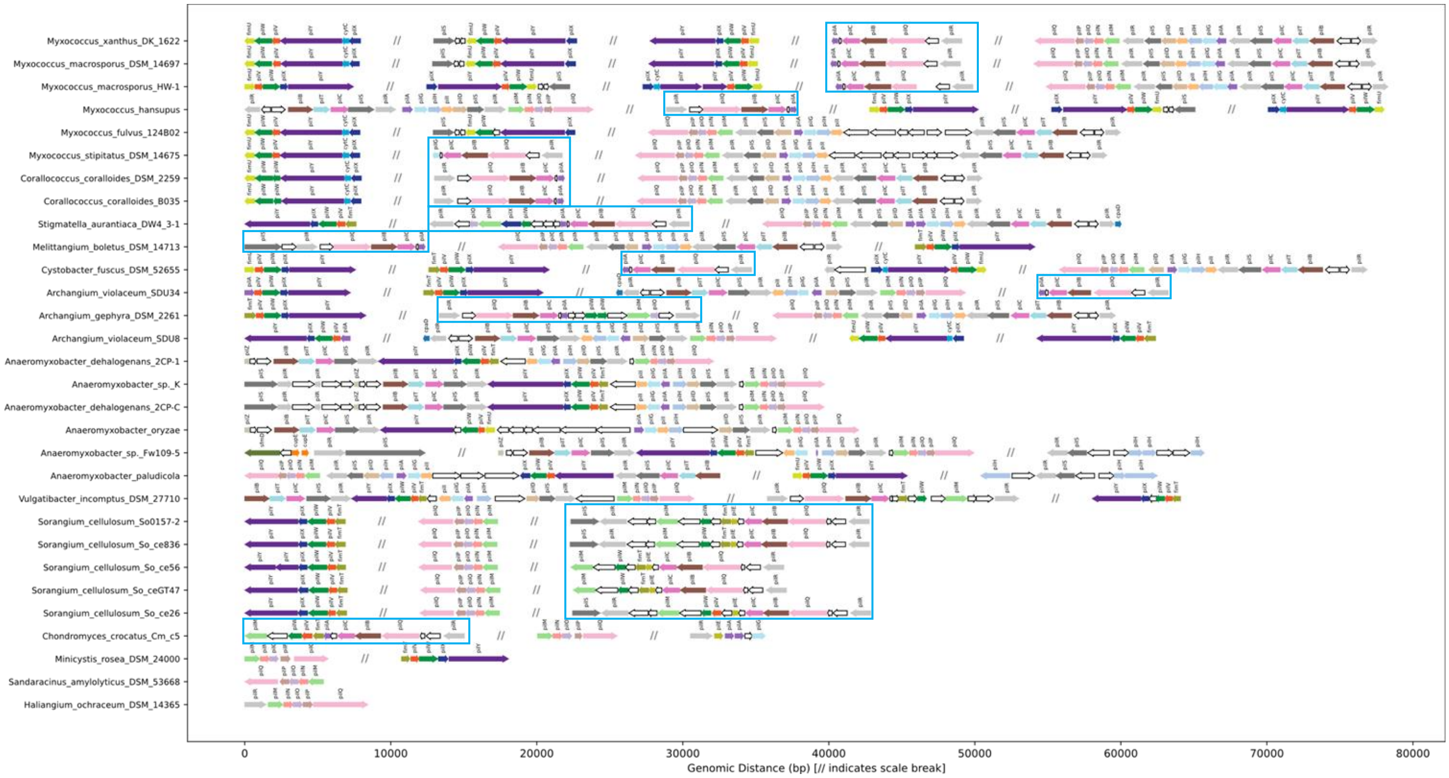

Supplementary Figure S2: Gene synteny of Type IVa pili (T4aP) major/minor pilin gene cluster and homologous type II secretion system (T2SS) components. Gene neighborhood plot showing the organization of T4aP genes across the complete genomes in the dataset. Homologous components of these genes in the T2SS are indicated in blue boxes.

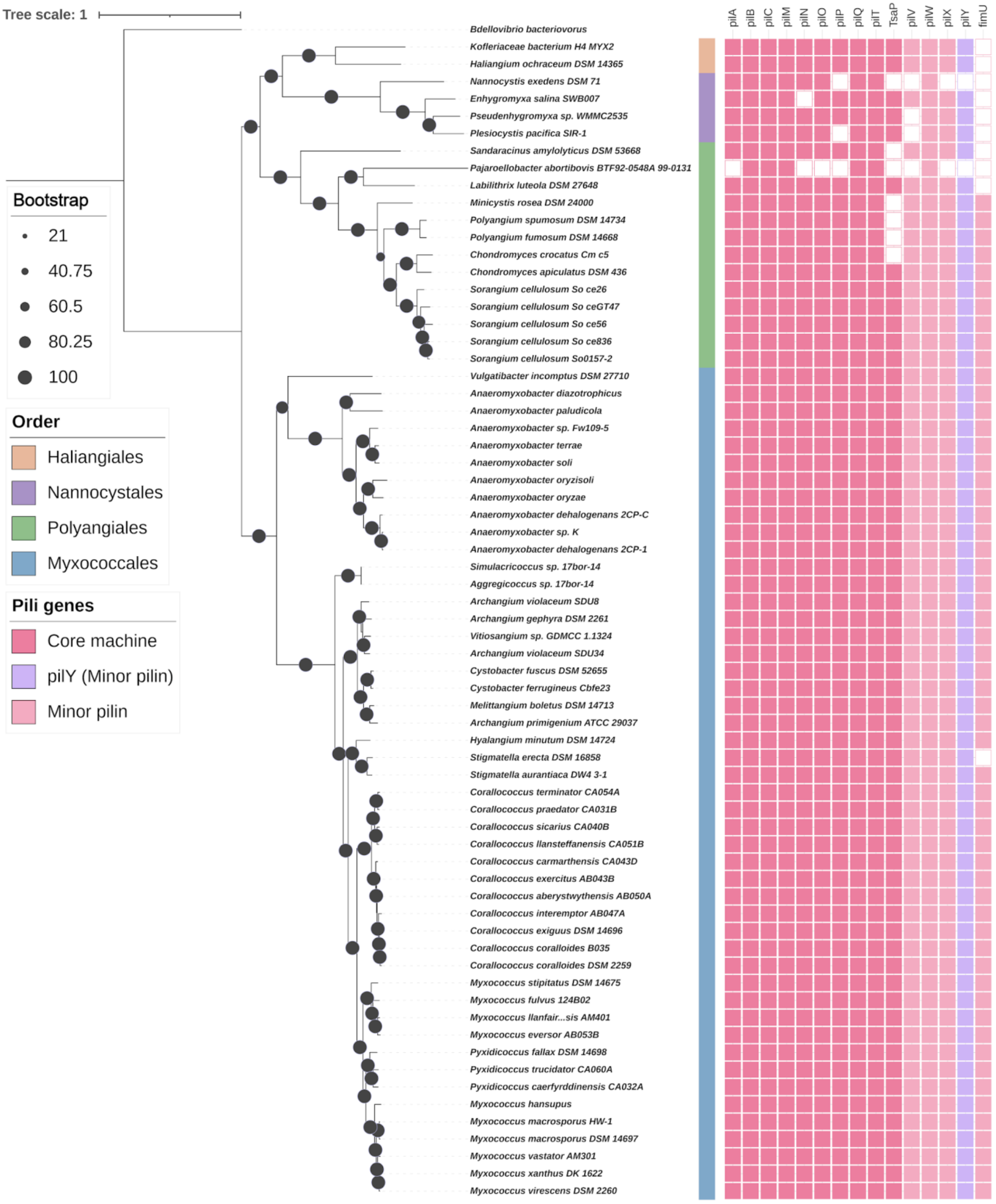

Supplementary Figure S3: Distribution of T4aP genes mapped onto the maximum likelihood Phylum Myxobacteriota phylogeny. ML tree corresponding to Figure 4, with the presence and absence of T4aP system genes indicated alongside each genome. Colored squares denote the detection of a given gene, whereas white squares indicate its absence.

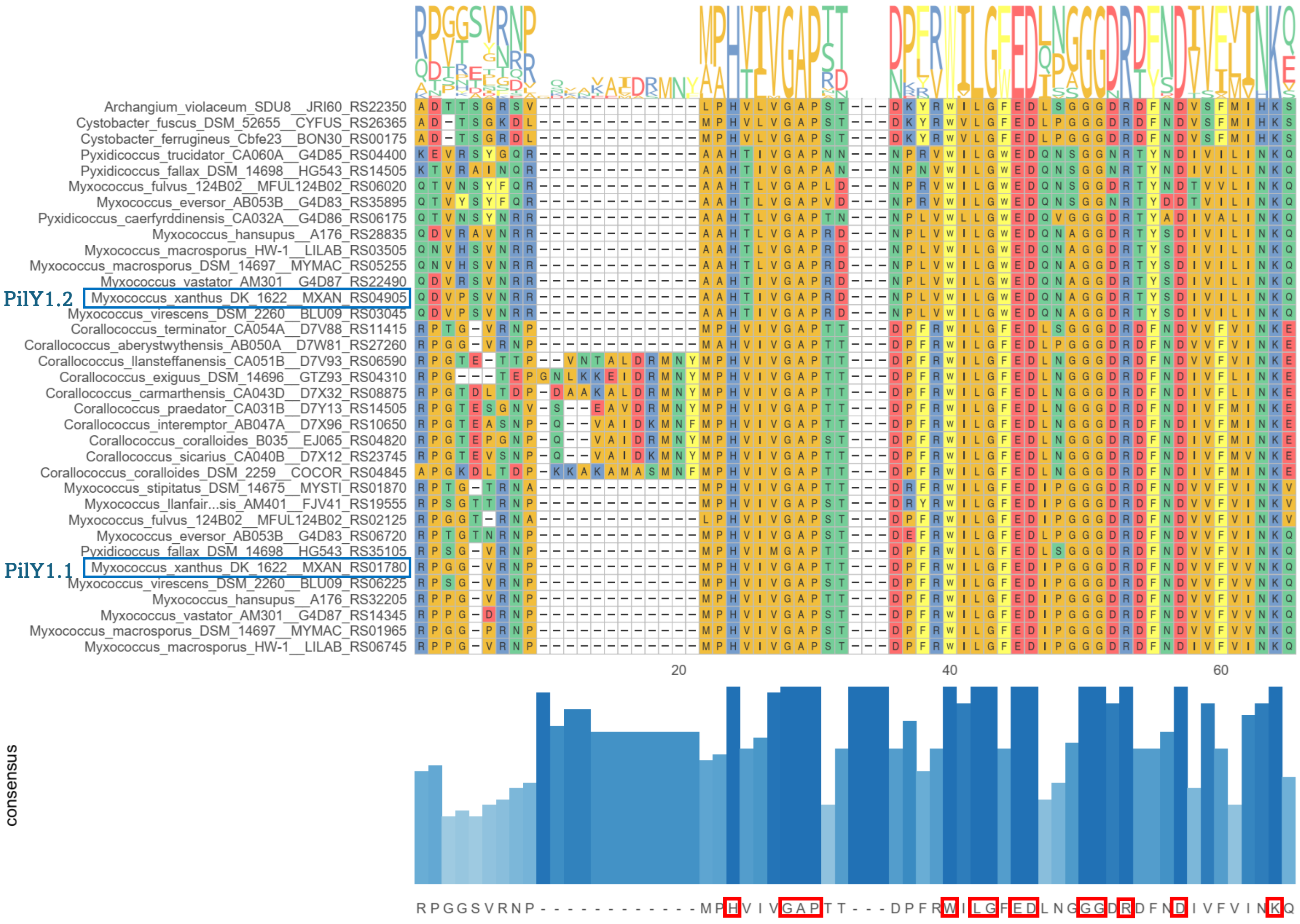

Supplementary Figure S4: Multiple sequence alignment of the DUF4114-domain containing region in Clade 1.1 and Clade 1.2 PilY1 proteins. The alignment highlights the conserved segment corresponding to the DUF4114-domain containing region in PilY1 proteins from both clades. PilY1.1 and PilY1.2 from *M. xanthus* are marked in blue. The region shown spans amino acids 458–507 in PilY1.1 and 489–538 in PilY1.2, containing the predicted DUF4114-domains as shown in Figure 1A (amino acids 481-501 for PilY1.1 and 507-532 for PilY1.2).

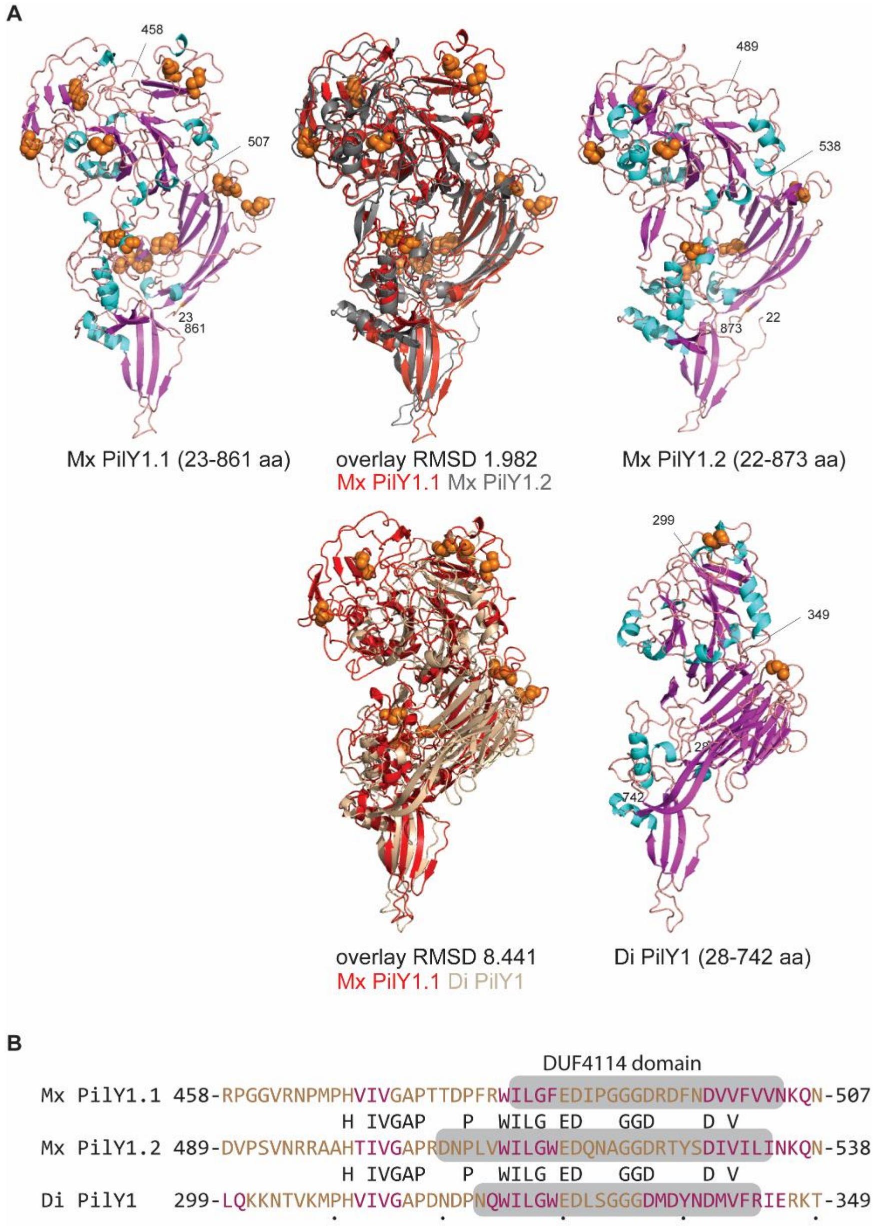

Supplementary Figure S5: Structural similarities between DUF4114-domain-containing PilY1 proteins. **(A)** AlphaFold models of the N-terminal regions of *M. xanthus* PilY1.1 and PilY1.2 (Mx) and *Desulfonema ishimotonii* PilY1 (Di PilY1, UniProt A0A401FQR6). The central panel shows overlay models of the indicated proteins, with corresponding RMSD values. Coordinates indicate the first and last amino acids depicted, as well as the DUF4114 domain-containing region, as shown in (B). Beta-Sheets are colored violet, α-helices turquoise, and unstructured regions gold. Disulfide bridges are represented as orange spheres. **(B)** Sequence alignment of the DUF4114 domain-containing region in *M. xanthus* PilY1.1 and PilY1.2 and *D. ishimotonii* PilY1. Violet residues indicate amino acids involved in β-sheets. The grey-shaded region corresponds to the predicted DUF4114 domain.

Supplementary Table S1: Sequence and structural similarities among PilY1 proteins.
Top table: Amino acid identities and similarities (%) between the β-propeller domains of the fifteen PilY1 proteins shown in Figure 1. **Middle table:** Amino acid identities and similarities (%) between the full-length PilY1 proteins from Figure 1. **Bottom table:** Root mean square deviation (RMSD) values obtained by superimposing the AlphaFold models of the fifteen PilY1 proteins in a one-to-one pairwise manner. The pTM scores of the corresponding AlphaFold models are listed in the second-to-last row. RMSD values calculated by superimposing each AlphaFold model against the experimentally determined structure 3HX6 are shown in the last row. These values are also displayed in Figure 1 for reference; their inclusion here facilitates direct comparison. All values are color-coded to enhance visual interpretation.

Supplementary Table S2: Distribution of PilY1 across the analyzed genomes. List of all organisms included in the dataset, indicating the number of PilY1 proteins identified in each genome and their corresponding phylogenetic clade and type assignments.

Supplementary Table S3: Comprehensive annotation of all detected PilY1 proteins. List of all identified PilY1 proteins across the dataset, including their assigned phylogenetic clade and PilY type. For each protein, the table provides sequence length, total number of cysteine residues, detected position of the K02674 profile, presence or absence of the DUF4114 domain, and detection of functional features including signal peptide, RGD motif, MIDAS motif, and Ca^{2+}-binding motifs.
